# Effect of a real-time automatic nosocomial infection surveillance system on hospital-acquired infection prevention and control

**DOI:** 10.1186/s12879-022-07873-7

**Published:** 2022-11-16

**Authors:** Ruiling Wen, Xinying Li, Tingting Liu, Guihong Lin

**Affiliations:** Department of Infection Management, Huizhou First People’s Hospital, No. 20, Sanxin South Road, Jiangbei, Huicheng District, Huizhou, Guangdong 516003 People’s Republic of China

**Keywords:** Hospital-acquired infections, Multidrug resistant organisms, Infections surveillance, Infection control

## Abstract

**Background:**

The systematic collection of valid data related to hospital-acquired infections (HAIs) is considered effective for nosocomial infection prevention and control programs. New strategies to reduce HAIs have recently fueled the adoption of real-time automatic nosocomial infection surveillance systems (RT-NISSs). Although RT-NISSs have been implemented in some hospitals for several years, the effect of RT-NISS on HAI prevention and control needs to be further explored.

**Methods:**

A retrospective, descriptive analysis of inpatients from January 2017 to December 2019 was performed. We collected hospital-acquired infection (HAI) cases and multidrug resistant organism (MDRO) infection cases by traditional surveillance in period 1 (from January 2017 to December 2017), and these cases were collected in period 2 (from January 2018 to December 2018) and period 3 (from January 2019 to December 2019) using a real-time nosocomial infection surveillance system (RT-NISS). The accuracy of MDRO infection surveillance results over the 3 periods was examined. The trends of antibiotic utilization rates and pathogen culture rates in periods 2 and 3 were also analysed.

**Results:**

A total of 114,647 inpatients, including 2242 HAI cases, were analysed. The incidence of HAIs in period 2 was significantly greater than that in period 1 (2.28% vs. 1.48%, χ^2^ = 61.963, p < 0.001) and period 3 (2.28% vs. 2.05%, χ^2^ = 4.767, p = 0.029). The incidence of five HAI sites, including respiratory infection, urinary tract infection (UTI), surgical site infection (SSI), bloodstream infection (BSI) and skin and soft tissue infection, was significantly greater in period 2 compared with period 1 (both p < 0.05) but was not significantly different from that in period 3. The incidence of hospital-acquired MDRO infections in period 3 was lower than that in period 2. The identification of MDRO infection cases using the RT-NISS achieved a high level of sensitivity (Se), specificity (Sp), positive predictive value (PPV) and negative predictive value (NPV), especially in period 3 (Se = 100%, Sp = 100%, PPV = 100% and NPV = 100%).

**Conclusion:**

The adoption of a RT-NISS to adequately and accurately collect HAI cases is useful to prevent and control HAIs. Furthermore, RT-NISSs improve accuracy in MDRO infection case reporting, which can timely and accurately guide and supervise clinicians in implementing MDRO infection prevention and control measures.

## Background

Hospital-acquired infections (HAIs), also known as nosocomial infections (NIs), are currently one of the most important challenges for modern medicine [1, 2]. Patients with HAIs might have prolonged hospital stays and high mortality, thus not only threatening the safety of patients but also causing a significant waste of social and economic resources, representing an important public health problem threatening human health [3–6]. Nosocomial infection surveillance is an important basis for controlling the occurrence and development of HAIs [6–10]. Hospitals should detect HAI cases and outbreaks of HAIs in a timely manner, analyse causes and take effective prevention and control measures. Timely reporting of nosocomial infection cases is of great significance for preventing nosocomial infection outbreaks and improving the quality of hospital management. However, due to the wide coverage and complexity of HAIs, a large amount of data needs to be analysed statistically, and traditional manual surveillance is inaccurate and inefficient and has been unable to meet the actual needs of HAI management [9]. The real-time automatic nosocomial infection surveillance system (RT-NISS) is a real-time, automatic and effective monitoring system that has been developed methodologically and practically in a stepwise manner and is a reliable surveillance intelligent information technology tool for infection control physicians to systematically valuate HAI information. A RT-NISS enhances case-finding efficiency by automatically and systematically selecting patients with the highest HAI probability. Once infection control physicians identify HAI cases, they can take strategies and measures to control HAI cases and accumulate experience to prevent subsequent HAI events [8, 11]. MDROs easily lead to nosocomial transmission and nosocomial cluster infection events. Thus, timely and accurate monitoring of MDROs is necessary. As the previous study showed, MDROs outbreaks controlled within a short time by RT-NISS early prewarning and infection control physicians’ immediate and effective control measures [12]. Therefore, the adoption of RT-NISS is one of the core components of infection prevention and control programs for modern medicine.

RT-NISS have been implemented in some hospitals for several years, but it remains an open question whether the use of a RT-NISS is effective in the reduction of nosocomial infections. Our study aimed to describe and analyse the effect of a RT-NISS on HAI prevention and control using surveillance data for a 3-year period from 2017 to 2019.

## Methods

### Patient population and study design

**Fig. 1:**
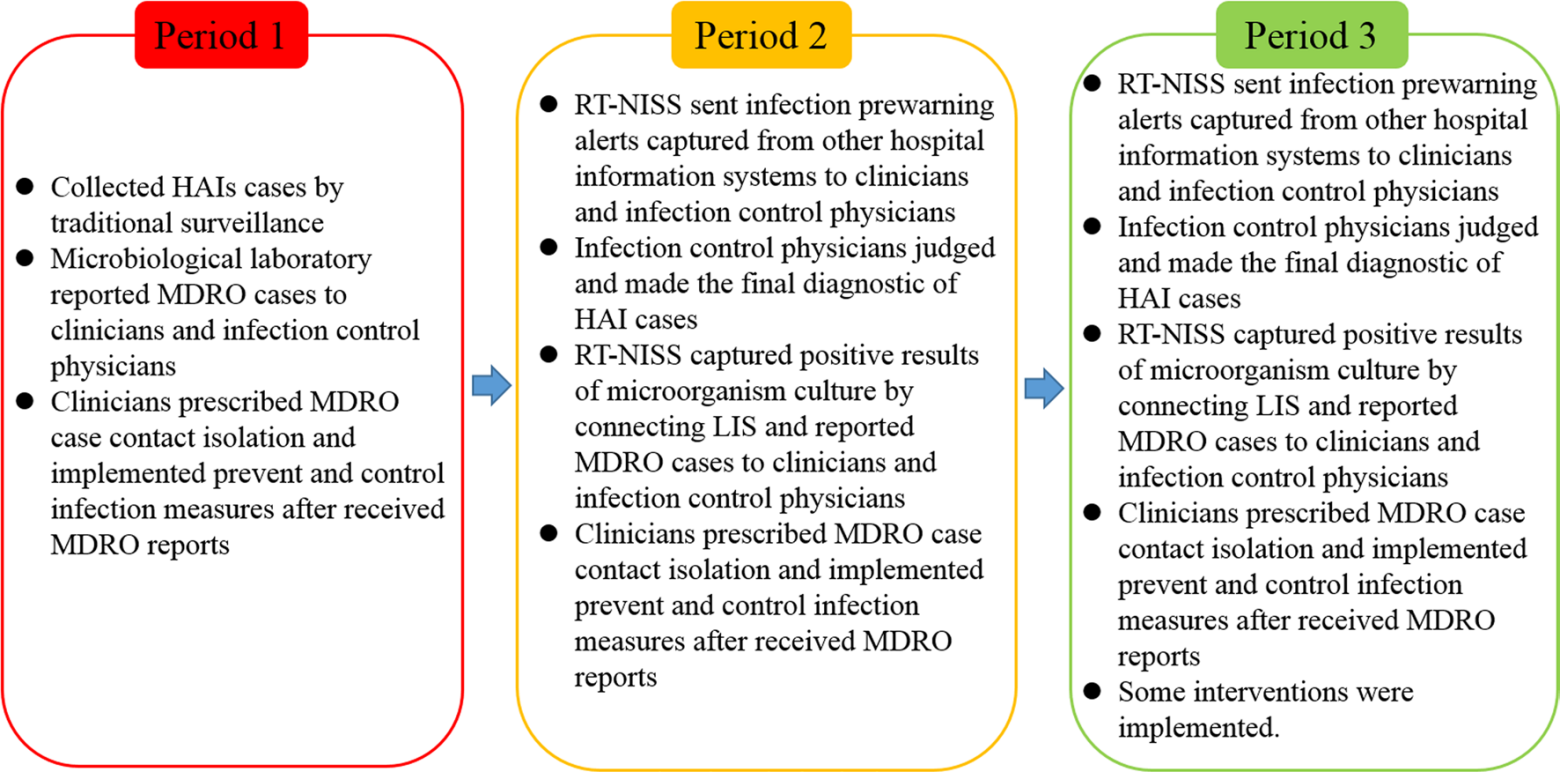
Study design. From January 1, 2017 to December 2017 (period 1), HAIs and MDRO cases were collected by traditional surveillance. The adoption of a RT-NISS to monitor HAIs and MDROs was implemented in period 2 (January 2018–December 2018). The adoption of a RT-NISS coupled with some infection-control interventions was implemented in period 3 (January 2019–December 2019)

We conducted a retrospective study of all patients admitted to Huizhou First Peoples’ Hospital (HZFH) from 2017 to 2019. Patients were categorized into three groups (Fig. 1): the first group (period 1) included patients admitted to HZFH from January 2017 to December 2017. We used traditional surveillance to collect hospital-acquired infection (HAI) cases and multidrug resistant organism (MDRO) infection cases. The second group (period 2) and the third group (period 3) included all patients admitted to HZFH from January 2018 to December 2018 and January 2019 to December 2019, respectively. In these two groups, we collected information regarding HAI cases, MDRO cases, the utilization rate of antibiotics and the rate of pathogen culture using the real-time nosocomial infection surveillance system (RT-NISS). In period 1, clinicians and infection control physicians obtained MDRO cases information entirely based on microbiology laboratory physicians’ daily reporting. In period 2 and 3, no longer dependent on microbiology laboratory physicians, the RT-NISS judged MDROs by capturing the original data from laboratory information system (LIS) and sent the MDROs information to clinicians and infection control physicians every day. Finally, all of the MDRO infection cases of 3 periods were peer-reviewed by senior infection control physicians to examine the accuracy of the surveillance results of the 3 periods.

**Fig. 2:**
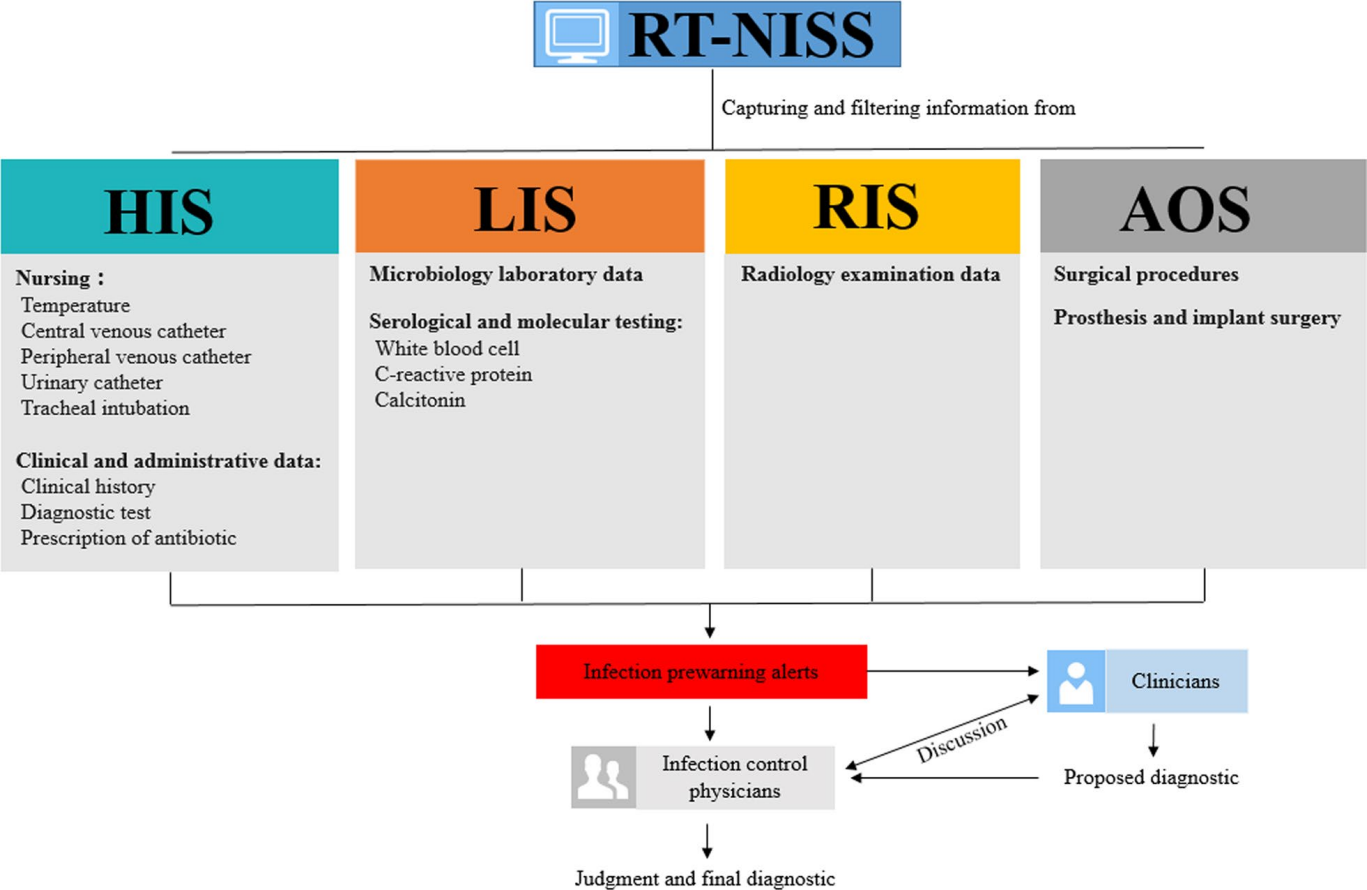
The infection surveillance operational process of the RT-NISS

Traditional surveillance is a time-consuming manual inspection that is mainly based on daily assessment of laboratory results and drug prescriptions from the pharmacy service, regularly visiting patients’ clinical histories and routinely receiving information on HAI cases reported by clinicians. The RT-NISS (Xinglin Technology, Hangzhou, China) used in our study was systematically applied to monitor, diagnose, and control HAIs under the strict supervision of infection control physicians. The RT-NISS selects patients with the highest HAI probability by connecting other hospital information systems, including the hospital information system (HIS), LIS, radiology information system (RIS) and anaesthesia operation system (AOS), and sets a filter to acquire the necessary infection information (Fig. 2). The RT-NISS was activated, captured patients’ infection information from other hospital information systems and provided new HAI alerts at 2:00 am every day. Infection control physicians and clinicians would obtain infection information and make judgment and final diagnostic by using RT-NISS.

### Definitions

Hospital-acquired infections (HAIs) were defined according to the Chinese NI diagnosis criterion published by Ministry of Public Health in 2001 [13]: Those infections occurring 48 h after hospital admission and have no evidence of subclinical infection at the time of admission were defined as HAIs. HAIs are classified as respiratory infection, urinary tract infection (UTI), gastrointestinal tract infection, surgical site infection (SSI), bloodstream infection (BSI), skin and soft tissue infection and other infections according to the specific infection site. MDROs were defined as those bacteria that were resistant to at least 3 antimicrobial classes, including extended-spectrum beta-lactamase (ESBL)-producing Gram-negative bacilli (mainly *Escherichia coli* and *Klebsiella pneumoniae*), methicillin-resistant *Staphylococcus aureus* (MRSA), carbapenem-resistant *Acinetobacter baumannii* (CRAB), carbapenem-resistant *Pseudomonas aeruginosa* (CRPA), carbapenem-resistant *Enterobacterales* (CRE) and vancomycin-resistant enterococci (VRE).

### Statistical methods

The following parameters were assessed for MDRO cases: TP = true positive (the collected positive case is truly positive), FP = false positive (negative case is classified as positive), FN = false negative (positive case is classified as negative), TN = true negative (the collected negative case is truly negative), Se = sensitivity (the proportion of positive cases correctly classified), Sp = specificity (the proportion of negative cases correctly collected), PPV = positive predictive value (the proportion of positive predictions that are correct) and NPV = negative predictive value (the proportion of negative predictions that are correct). The χ^2^-test was used for the comparison of categorical variables. All statistical analyses were performed by using SPSS 22.0 software (IBM SPSS), and 2-tailed p values < 0.05 were considered statistically significant.

## Results

### A comparison between traditional surveillance and RT-NISS

We used traditional surveillance to collect 34,197 patients, including 505 (1.48%) HAI cases in period 1. Of these HAI patients, 318 (0.93%) suffered respiratory infection, 84 (0.25%) suffered UTI, 25 (0.07%) suffered GTI, 30 (0.09%) suffered SSI, 48 (0.14%) suffered BSI and 40 (0.12%) suffered skin and soft tissue infection. In period 2, 38,028 patients, including 866 (2.28%) HAI cases, were collected from the RT-NISS. Of these HAI patients, 526 (1.38%) suffered respiratory infection, 219 (0.58%) suffered UTI, 31 (0.08%) suffered GTI, 55 (0.14%) suffered SSI, 86 (0.23%) suffered BSI and 80 (0.21%) suffered skin and soft tissue infection. The incidence of HAIs in period 2 was significantly greater than that in period 1 (χ^2^ = 61.963, p < 0.001). With the exception of GTI, the incidences of different HAI sites, including respiratory infection, UTI, SSI, BSI and skin and soft tissue infection, in period 2 were also significantly greater than those in period 1 (Table 1). The incidence of hospital-acquired MDROs in period 1 was 0.56% (191 cases). However, in period 2, the incidence was 0.52% (197 cases), and no significant difference was noted compared with period 1. These results demonstrated that the adoption of RT-NISS improved the detection rate of HAI cases and eliminated the serious underreporting phenomenon of traditional surveillance.

**Table 1:** Comparative results of HAIs reported by traditional surveillance during period 1 and RT-NISS in periods 2 and 3

Infection type	Period 1 (n = 34,197)	Period 2 (n = 38,028)	Period 3 (n = 42,422)	$\chi_{1}^{2}$	P_1_	$\chi_{2}^{2}$	P_2_
HAIs cases (n/%)	505 (1.48)	866 (2.28)	871 (2.05)	61.963	< 0.001	4.767	0.029
Respiratory infection	318 (0.93)	526 (1.38)	538 (1.27)	32.033	< 0.001	2.031	0.154
UTI	84 (0.25)	219 (0.58)	202 (0.48)	47.008	< 0.001	3.831	0.050
GTI	25 (0.07)	31 (0.08)	41 (0.10)	0.164	0.685	0.513	0.474
SSI	30 (0.09)	55 (0.14)	87 (0.21)	5.232	0.022	3.847	0.050
BSI	48 (0.14)	86 (0.23)	89 (0.21)	7.155	0.007	0.247	0.619
Skin and soft tissue infection	40 (0.12)	80 (0.21)	78 (0.18)	9.470	0.002	0.719	0.397
Hospital-acquired MDROs (n/%)	191 (0.56)	197 (0.52)	173 (0.41)	0.552	0.457	5.322	0.021

*UTI* urinary tract infection, *GTI* gastrointestinal tract infection, *SSI* surgical site infection, *BSI* bloodstream infection, $\chi_{1}^{2}$ and P1 for comparison between period 1 and period 2, $\chi_{2}^{2}$ and P2 for comparison between period 2 and period 3

### The effect of RT-NISS on HAI prevention and control

In period 3, we used RT-NISS to collect 42,422 patients, including 871 (2.05%) HAI cases. Of these HAI patients, 538 (1.27%) suffered respiratory infection, 202 (0.48%) suffered UTI, 41 (0.10%) suffered GTI, 87 (0.21%) suffered SSI, 89 (0.21%) suffered BSI and 78 (0.18%) suffered skin and soft tissue infection. The incidence of HAIs in period 3 was significantly lower than that in period 2 (χ^2^ = 4.767, p = 0.029). The incidences of four hospital-acquired infection sites, including respiratory infection, UTI, BSI and skin and soft tissue infection, in period 3 were slightly lower than those in period 2, but the difference was not statistically significant. The incidence of hospital-acquired MDROs was 0.41% (173 cases) in period 3, which was significantly lower than that in period 2 (χ^2^ = 5.322, p = 0.021). After RT-NISS implementation in period 2, we collected HAI cases and analysed the risk factors for HAIs. Then, we took these risk factors into consideration and implemented some interventions that could prevent and control HAI events in period 3. As shown, the adoption of RT-NISS coupled with the implemented interventions in period 3 could avoid HAI events to some extent and reduce the HAI rate.

### RT-NISS improves accuracy in MDRO case reporting

**Fig. 3:**
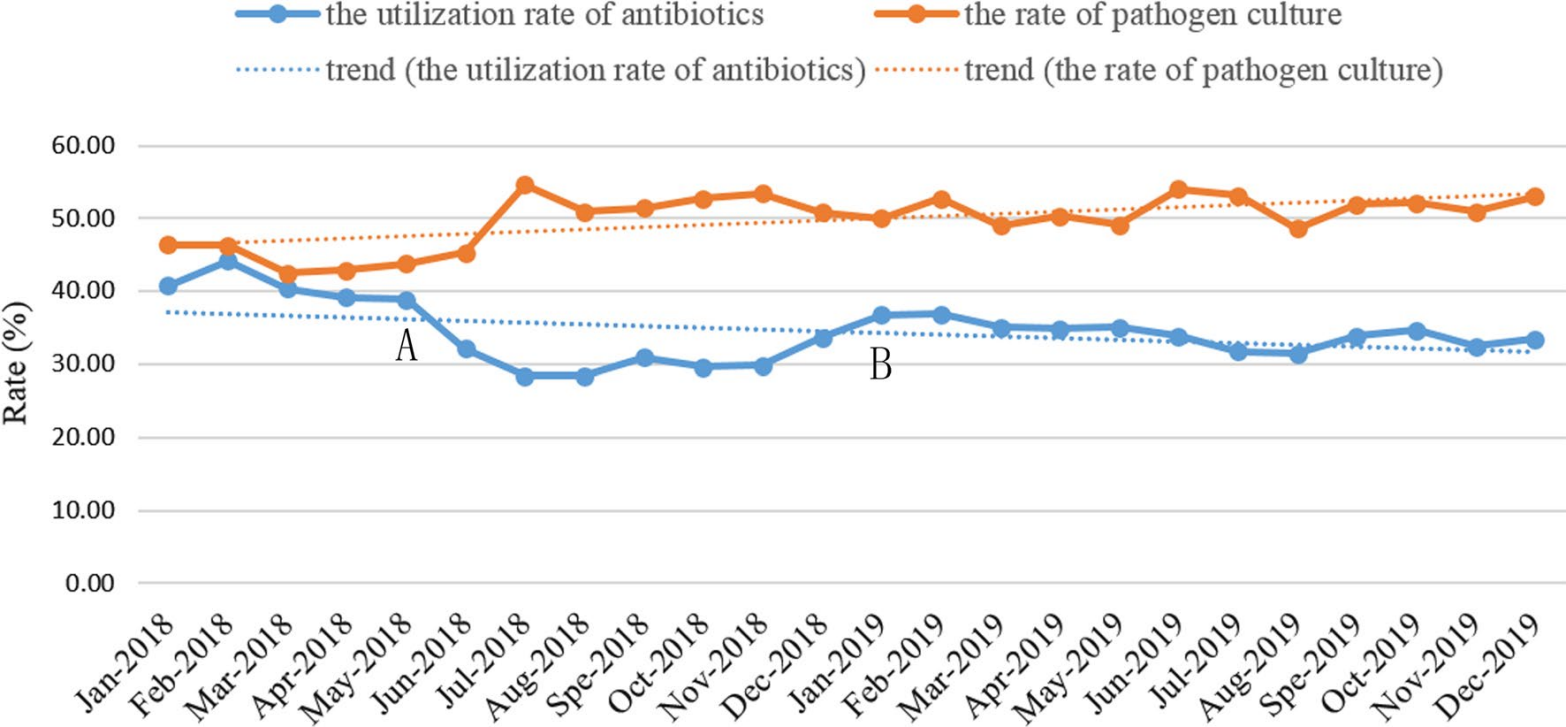
Monthly utilization rate of antibiotics and rate of pathogen culture in HZFH from January 2018 to December 2019. **A** intervention implemented: encouraged and trained clinicians to appropriately use antibiotics and timely pathogen culture. **B** intervention implemented: special education and harsh economic measures were implemented for those wards didn't implement prevention and control measures for MDROs and/or inappropriate administered antibiotics and/or under standard rate of pathogen culture

To further investigate the effect of the adoption of RT-NISS on certain HAI prevention and control programs, such as MDRO infection case surveillance and control, we examined the accuracy of MDRO infection case reporting by traditional surveillance and RT-NISS. As shown in Table 2, high numbers of FPs and FNs in both varieties of MDROs, including ESBL, MRSA, CRAB, CRPA and CRE, were noted in period 1. In periods 2 and 3, the number of FPs and FNs of both varieties of MDROs was significantly reduced. In particular, in period 3, these values were reduced to 0. As shown in Table 3, the Se, Sp, PPV and NPV of both 5 different varieties of MDROs, including ESBL, MRSA, CRAB, CRPA and CRE, in period 1 were significantly lower than those in period 2 and period 3. Furthermore, the Se, Sp, PPV and NPV of both varieties of MDROs in period 3 were all improved to 100%. In addition, no VRE cases were reported from 2017 to 2019 in HZFH. After the RT-NISS implementation in period 2, we collected causes of mistaken reports of MDRO infection cases, and we fixed the mistakes of the RT-NISS operation in period 3. In addition, we also monitored the utilization rate of antibiotics and the rate of pathogen culture by RT-NISS. As shown in Fig. 3, the trend of the utilization rate of antibiotics from January 2018 to December 2019 slightly decreased, whereas the rate of pathogen culture slightly improved.

**Table 2:** Respective comparisons of MDROs reported by traditional surveillance in period 1 and the RT-NISS in periods 2 and 3 by senior infection control physicians

Group	ESBL				MRSA				CRAB				CRPA				CRE			
	TP	FP	FN	TN	TP	FP	FN	TN	TP	FP	FN	TN	TP	FP	FN	TN	TP	FP	FN	TN
Period 1	325	82	192	562	85	91	21	114	44	23	15	106	25	52	19	236	0	0	29	1050
Period 2	388	14	13	557	101	0	19	139	50	0	7	119	38	24	5	228	0	0	14	944
Period 3	392	0	0	614	81	0	0	134	48	0	0	100	26	0	0	243	15	0	0	991

*FP* true positive, *FP* false positive, *FN* false negative, *TN* true negative

**Table 3:** Accuracy examination results of traditional surveillance in period 1 and RT-NISS in periods 2 and 3, for a 95% confidence interval

MDRO type	Group	Se	Sp	PPV	NPV
ESBL	Period 1	62.86 (58.61–66.92)	87.27 (84.47–89.63)	79.85 (75.68–83.46)	74.54 (71.31–77.52)
	Period 2	96.75 (94.54–98.10)	97.55 (95.93–98.54)	96.52 (94.24–97.92)	97.72 (96.14–98.66)
	Period 3	100 (99.03–100)	100 (99.38–100)	100 (99.03–100)	100 (99.38–100)
MRSA	Period 1	80.19 (71.61–86.66)	55.61 (48.77–62.25)	48.30 (41.03–55.64)	84.44 (77.38–89.59)
	Period 2	84.17 (76.59–89.63)	100 (97.31–100)	100 (96.34–100)	87.97 (81.98–92.16)
	Period 3	100 (95.47–100)	100 (97.21–100)	100 (95.47–100)	100 (97.21–100)
CRAB	Period 1	74.58 (62.21–83.95)	82.17 (74.66–87.81)	65.67 (53.73–75-91)	87.60 (80.55–92.34)
	Period 2	87.72 (76.75–93.92)	100 (96.87–100)	100 (92.87–100)	94.44 (88.97–97.28)
	Period 3	100 (92.59–100)	100 (96.30–100)	100 (92.59–100)	100 (96.30–100)
CRPA	Period 1	56.82 (42.23–70.32)	81.94 (77.09–85.95)	32.47 (23.06–43.54)	92.55 (88.66–95.18)
	Period 2	88.37 (75.52–94.93)	90.48 (86.22–93.52)	61.29 (48.85–72.42)	97.85 (95.07–99.08)
	Period 3	100 (87.13–100)	100 (98.44–100)	100 (87.13–100)	100 (98.44–100)
CRE	Period 1	0 (0–11.70)	100 (99.64–100)	0 (0)	97.31 (96.16–98.12)
	Period 2	0 (0–21.53)	100 (99.59–100)	0 (0)	98.54 (97.56–99.13)
	Period 3	100 (79.61–100)	100 (99.61–100)	100 (79.61–100)	100 (99.61–100)

*Se* sensitivity, *Sp* specificity, *PPV* positive predictive value, *NPV* negative predictive value

**Table 4:** The checklist of interventions implemented in period 3

Interventions
• Quality management tools were used to analyze the risk factors for certain infections in high infection wards or high infection sites (e.g., urinary tract infection, sepsis, pneumonia), and appropriate improvement measures not only but including supervision of hand hygiene, strengthening environmental hygiene and increasing environmental hygiene monitoring were formulated
• Implemented MDRO cases transmission preventions: especially hand hygiene, environmental hygiene, isolation in combination with personal protective equipment
• Sufficient trainings (e.g., about adequate hand hygiene, environment hygiene, prevention and control knowledge of infections and so on) were provided to healthcare workers including physicians, nurses and cleaners
• The utilization rate of antibiotics and the rate of pathogen culture monitored by RT-NISS. Reported monthly utilization rate of antibiotics and rate of pathogen culture in each ward • Performance reward or economic measures was used to encourage outstanding infection control management wards and restrain terrible infection control management situation
• Enhanced cooperation between infection control physicians and healthcare workers for preventing and controlling HAIs

## Discussion

Surveillance of HAIs is a fundamental and essential aspect of infection prevention and control programs [6–10]. Traditional surveillance is a manual time-consuming infection case-finding and reporting program that involves infection control physicians looking over lists of positive infection information of pathogen culture and patient histories daily and HAI case reports by clinicians. In this study, the results showed low infection incidences of HAIs (1.48%) and certain infection sites, including respiratory infection (0.93%), UTI (0.25%), SSI (0.09%), BSI (0.14%) and skin and soft tissue infection (0.12%), reported by traditional manual surveillance methods in period 1. These low reported infection incidences echo the inaccurate report demonstrated by other studies [9, 12]. Seifi’s study presented the results of under- and overreporting of HAIs by traditional surveillance [9]. Many hospitals have a large number of beds but insufficient numbers of infection control physicians, and infection control physicians need to collect large amounts of data daily, which will result in false negative infection reports. Many cases are excessive, or unnecessary antibiotic usage will lead to false-positive infection findings. In addition, many cases are not ordered for pathogen culture to cause false negative infection reports. Moreover, the lack of knowledge regarding complicated infection cases of clinicians will lead to underreporting and mistaken reporting of infection events. Given their high workloads and need for multitasking, clinicians can make mistakes and lack sufficient time to report infection cases. All these factors make this type of manual surveillance unreliable and lack accuracy. There is no doubt that this frustrating situation would have serious consequences, including absent treatment for underreporting HAI patients, inappropriate treatment of false-positive infection patients, transmission of pathogens from HAI patients to other uninfected patients, and many HAIs and outbreaks that are not monitored and uncontrolled. Therefore, this predicament has catalysed the development and implementation of RT-NISSs.

Many studies in the past have demonstrated accurate and efficient electronic automatic surveillance systems [11, 12, 14–16]. Our analysis showed higher infection incidences of HAIs and certain infection sites, including respiratory infection, UTI, SSI, BSI and skin and soft tissue infection, reported by RT-NISS (both in period 2 and period 3) compared with those reported by traditional surveillance in period 1 (both p < 0.05), which is consistent with the accurate surveillance results based on RT-NISSs reported in other studies. Mingmei’s study presented the sensitivity and specificity of a RT-NISS with an approximately 200-fold time-saving capacity compared with manual surveys [12]. Studies in this field are more concerned with defects by traditional manual surveillance and the virtues by RT-NISSs on the surveillance of all HAIs. However, few analyses have evaluated the use of RT-NISSs for surveillance of different types of MDRO or have only focused on one type of MDRO at a time [17, 18]. In the present study, we demonstrated a more accurate surveillance result of MDRO reports by the RT-NISS in both periods 2 and 3 compared with traditional surveillance in period 1 (Tables 2, 3). In period 1 of the present study, clinicians and infection control physicians obtained MDRO case information based on microbiology laboratory physicians’ daily reporting. However, microbiology laboratory physicians underreported MDRO cases or reported cases in an untimely manner due to their high workload, that led to the high FN numbers of both kinds of MDROs. Furthermore, there weren’t sufficient trainings for microbiology laboratory physicians led to erroneous judgement or mis-classification of MDROs that made the FP and FN numbers high in the period 1. We present the results of a lower number of FPs and FNs and higher Se, Sp, PPV and NPV of 5 different varieties of MDROs, including ESBL, MRSA, CRAB, CRPA and CRE, since RT-NISS implementation. Compared with period 1, the MDRO reporting process and surveillance changed, and the MDRO information and data became reliable and accurate. MDROs are the main pathogenic bacteria of SSI, UTI, BSI and pneumonia, and these types of nosocomial infections are intractable and complex and the factor of high mortality for patients. Moreover, MDROs easily lead to nosocomial transmission and nosocomial cluster infection events [19–21]. Thus, timely and accurate monitoring of MDROs is an important targeted monitoring project for infection prevention and control programs [12]. The results of the present study about MDROs surveilled by RT-NISS could benefit and inform targeted prevention and control programs.

Another strength of our study was the investigation of the effect of the daily implementation of RT-NISS on HAI surveillance. After RT-NISS implementation in period 2, we analysed the long-term data collected by RT-NISS and acquired comprehensive knowledge about HAI risk factors. Then, we implemented some interventions (Table 4). And for those wards didn’t implement prevention and control measures for MDROs and/or inappropriately administered antibiotics and/or without standard pathogen culture, we reported monthly utilization rate of antibiotics and rate of pathogen culture in each ward to remain them, provided special education for the healthcare workers and harsh economic measures were implemented for the serious situation. The incidences of HAIs and hospital-acquired MDROs in period 3 were significantly lower than those in period 2 (0.41% vs. 0.52%, p = 0.021). These results demonstrated that the adoption of RT-NISS coupled with the implemented interventions could reduce the rate of HAIs. In addition, regarding the results of the MDROs reported by RT-NISS in period 2, we further found 3 mistakes associated with the use of the RT-NISS. 1. There were 24 other kinds of muti-drug resistance Pseudomonas species misclassified as CRPA resulted in 24 FP of CRPA. 2. 14 CRE misclassified as ESBL resulted in 14 FP of ESBL and 14 FN of CRE. 3. 13 ESBL, 19 MRSA, 7 CRAB and 5 CPPA were under-reported because of the LIS connection fails by RT-NISS. Based on those above mistakes, we checked and corrected different varieties of MDROs’ rule definition of RT-NISS, we also required RT-NISS engineers regularly check the connection with other hospital information systems and timely fixed the connection fails in period 3. After modifying the rule definition and timely correcting the data input, the number of FPs and FNs of 5 different varieties of MDROs, including ESBL, MRSA, CRAB, CRPA and CRE, in period 3 was reduced to 0, and the Se, Sp, PPV and NPV of 5 different varieties of MDROs in period 3 were both improved to 100%.

Our study still has a limitation. Due to all patients’ information in the period 1 (the manual surveillance phase) were collected only by manual surveillance, it is difficult to obtain all of the sufficient information of large number of patients. Thus, would the composition of the patient population or the specialty of the patients affect the individual infection types in 3 periods of the present study is not sure.

## Conclusion

With an increasing number of studies in this field demonstrating the accuracy and efficiency of RT-NISS on HAI surveillance, health care authorities increasingly demand the installation and daily use of RT-NISSs as a part of quality management. Our data showed that RT-NISS could adequately and accurately collect HAI cases, which is in agreement with other studies [12, 16, 22]. Furthermore, RT-NISS improves the accuracy of MDRO infection case reports, and the adoption of a RT-NISS coupled with intervention implementations can reduce the infection incidences of HAIs and MDROs.

## Data Availability

The datasets analysed during the current study are not publicly available due to privacy or ethical restrictions but are available from the corresponding author on a reasonable request.

## Abbreviations

HAIs: Hospital-acquired infections
RT-NISS: Real-time automatic nosocomial infection surveillance system
MDROs: Multidrug resistant organisms
UTI: Urinary tract infection
SSI: Surgical site infection
BSI: Bloodstream infection
HIS: Hospital information system
LIS: Laboratory information system
RIS: Radiology information system
AOS: Anaesthesia operation system
TP: True positive
FP: False-positive
FN: False negative
TN: True negative
Se: Sensitivity
Sp: Specificity
PPV: Positive predictive value
NPV: Negative predictive value
ESBL: Extended-spectrum beta-lactamase producing Gram-negative bacilli
MRSA: Methicillin-resistant *Staphylococcus aureus*
CRAB: Carbapenem-resistant *Acinetobacter baumannii*
CRPA: Carbapenem-resistant *Pseudomonas aeruginosa*
CRE: Carbapenem-resistant *Enterobacterales*
VRE: Vancomycin-resistant enterococci
